# Learning, adopting, and sustaining the pharmacist-clinician role in clinical practice: a qualitative study among early adopters in the Netherlands

**DOI:** 10.1007/s11096-026-02150-y

**Published:** 2026-05-08

**Authors:** Kim-Lara Klerk-Bos, Marije P. Hennus, Marco Ehlert, Manon Kluijtmans, Toine C. G. Egberts, Ingeborg Wilting

**Affiliations:** 1 Department of Clinical Pharmacy, University Medical Center Utrecht, Utrecht, The Netherlands; 2 Pediatric Intensive Care, Wilhelmina Children’s Hospital, University Medical Center Utrecht, Utrecht, The Netherlands; 3 Division of Pharmacoepidemiology and Clinical Pharmacology, Faculty of Science, Utrecht Institute for Pharmaceutical Science, Utrecht University, Utrecht, The Netherlands; 4 Education Center, University Medical Center Utrecht, Utrecht, The Netherlands; 5 University College Utrecht, Utrecht University, Utrecht, The Netherlands

**Keywords:** Hospital pharmacist, Interprofessional, Pharmacist-clinician, Workplace learning

## Abstract

**Introduction:**

Hospital pharmacists are increasingly involved in direct patient care; however, little is known about how to learn, adopt, and sustain this pharmacist-clinician role in practice.

**Aim:**

This study aimed to examine the implementation of the pharmacist-clinician role by exploring the key factors that shape its learning, adoption, and sustainment in clinical practice.

**Method:**

Early adopters of the pharmacist-clinician role in Dutch hospitals were recruited through purposive sampling via professional networks and national outreach. A semi-structured interview was conducted with the respondents. Data were analysed inductively using thematic analysis and were subsequently interpreted using the Normalisation Process Theory (NPT) to investigate key factors in learning, adopting, and sustaining the pharmacist-clinician role.

**Results:**

A total of 21 Dutch pharmacists working in a hospital were included. Factors, grouped into four themes (Scope and role enactment of the pharmacist-clinician role, interprofessional collaboration as the driver for adoption, experiential workplace learning is more important than formal learning, and organisational conditions shape sustainment and scalability) described how the pharmacist-clinician role develops from local sense-making and interprofessional adoption to enactment through workplace learning. Although individual initiative enabled early uptake, the role remained highly context-dependent. An NPT-informed synthesis showed that long-term sustainment relied mainly on organisational support and structural embedding, and less on individual factors.

**Conclusion:**

Our findings imply that embedding the pharmacist-clinician role should be approached as an organisational effort rather than merely as an expansion of professional competencies. Its sustainable development requires deliberate implementation, clear role definition, and organisational support for embedding the role in everyday practice.

**Supplementary Information:**

The online version contains supplementary material available at 10.1007/s11096-026-02150-y.

## Impact statements

- This study shows that the pharmacist-clinician role lacks shared understanding, underscoring the need for clearer conceptualisation of its scope and responsibilities to enable more consistent implementation.
- Adoption and early development of the pharmacist-clinician role are driven by interprofessional collaboration, individual initiative, and experiential workplace learning.
- Long-term normalisation of pharmacist-clinician work requires organisational and systemic change, not educational reform alone.
- Strengthening pharmacist-clinician practice therefore requires action not only from educators and practitioners, but also from organisational leaders and policymakers to create the conditions for sustainable role enactment.

## Introduction

The tasks and responsibilities of hospital pharmacists have, over the past decades, evolved from primarily product-oriented towards a combination of product-oriented tasks and responsibilities in direct patient care. Traditional tasks mainly involve indirect patient care, such as drug dispensing, medication surveillance, compounding medication, and therapeutic drug monitoring. In contrast, pharmacist-clinician tasks focus on direct patient care, including participation in ward rounds, multidisciplinary team meetings, patient consultations, medication reviews, education of healthcare professionals, patients, and families, and medication and symptom management [1–3]. In this study, we use the term *pharmacist-clinician* to describe pharmacists engaged in direct patient care, including hospital pharmacists (in training) and those working in ambulatory care settings. We deliberately avoid the term *clinical pharmacist*, as it is used inconsistently across contexts and may create conceptual ambiguity. Existing literature shows that *clinical pharmacy* is broadly defined as both a professional practice and a field of research aimed at optimising the use of medicines, encompassing cognitive, managerial, and interpersonal activities across all stages of the medication-use process and across all healthcare settings [4, 5]. In contrast, the term *pharmacist-clinician* in this study is used more narrowly to emphasise the enactment of direct patient care within this broader domain. In this sense, pharmacist-clinician roles can be understood as a specific manifestation of clinical pharmacy practice, focusing on patient-facing activities and integration within multidisciplinary care.

Multiple studies have demonstrated the added value of pharmacist-clinicians in direct patient care by showing effects on improved health outcomes and increased efficiency [6–9]. Although these pharmacist-clinician initiatives have been associated with benefits for patients and interprofessional collaboration, the role remains inconsistently defined, and its implementation remains limited and uneven across countries [10, 11]

In the Netherlands, the national association of hospital pharmacists (NVZA) actively promotes the pharmacist-clinician role in their strategic plan and has incorporated it into the national residency training program [12]. However, despite this, the role remains partially and variably implemented in routine hospital practice [13]. Many initiatives are locally organised and highly individual-dependent; initiatives often diminish when initiators leave, reflecting fragile implementation and limited sustainment.

The existing literature on pharmacist-clinician work has largely focused on individual competencies, tasks, and role descriptions [16–17]. While this research has clarified the skills required for clinical pharmacy practice and the scope of pharmacist involvement in direct patient care, comparatively little attention has been paid to the contextual conditions that shape how the role is adopted and sustained in everyday hospital settings. In particular, organisational prerequisites and embedding processes remain underexplored, resulting in a limited understanding of the factors that enable sustainable role development despite growing policy ambitions and educational investments in direct patient care.

To address the current knowledge gap and complement the competency development perspective, we use Normalisation Process Theory (NPT) as a theoretical lens to evaluate the implementation of the pharmacist-clinical role as an innovation process. NPT is a sociological action theory that explains how new practices or roles become implemented and normalised in everyday practice by focusing on the work of individuals and collectives [18]. As healthcare changes often involve shifts in roles, responsibilities, workflows, and professional boundaries rather than discrete interventions, NPT is particularly well-suited to examining why some initiatives become routinely embedded while others remain fragile.

### Aim

This study aimed to examine the implementation of the pharmacist-clinician role by exploring the key factors that shape its learning, adoption, and sustainment in clinical practice.

## Method

This study is being reported according to the consolidated criteria for reporting qualitative research (COREQ) checklist [19].

### Study design, study participants and recruitment

We conducted a qualitative, explorative study using semi-structured interviews with pharmacists who have early adopted a pharmacist-clinician role in practice. Using the experiences of hospital pharmacists already having working experience in this role (early adopters), we explore how the role is enacted in everyday work, what enables its initiation and continuation, and how organisational conditions influence longer-term embedding.

Participants were pharmacists who work in a hospital in the Netherlands, self-reporting as having experience with clinical tasks in direct patient care. Participants were recruited using purposive sampling. We contacted 23 hospital pharmacists personally via email and conducted a general outreach via the Dutch Association for Hospital Pharmacists newsletter. A total of 21 participated of which 17 via the personal invitation and 4 via the general outreach. Selection was based on the following criteria:

1. Willingness to participate
2. Self-identifying as currently or in the past performing a pharmacist-clinician role in their organisation.

### Data collection

Semi-structured interviews were conducted between November and December 2024 by two researchers (ME, KL) using a structured topic guide (see Appendix 1). The interview was pre-tested in four pilot interviews and iteratively refined during data collection. For pragmatic reasons, participants could choose whether the interview was conducted via video call or face-to-face. The interviews were recorded using MS Teams or a recording device. The interviews lasted 45–60 min.

Although this study was exploratory, we aimed to conduct interviews until saturation was reached, meaning until no new themes were identified [20]. We also aimed to include at least two participants in each subgroup, and therefore continued recruitment for a small number of additional participants after saturation had been achieved to ensure adequate representation across subgroups.The interviews were transcribed verbatim using Amberscript® when conducted in person, or via Microsoft Teams® when conducted online. The generated transcripts were manually verified and pseudonymised by the researchers (ME, KL). Participants were assigned sequential identifiers (P01, P02, etc.) based on the chronological order of the interviews.

### Data analysis

The interview data were analysed in two sequential phases, combining an inductive thematic approach followed by a theory-informed deductive step. Both steps were critically discussed with the whole author team. The qualitative data were analysed following ‘Braun and Clarke’s six-step inductive thematic analysis framework using NVivo^©^ 14/15 [21].

As the inductive themes indicated that sustainment of the pharmacist-clinician role could not be sufficiently explained by individual attributes alone and appeared to be shaped by contextual and collective processes, we applied Normalisation Process Theory (NPT) to further explore sustainment.

NPT operationalises implementation work through four interrelated constructs that capture the sequential and overlapping forms of labour undertaken by stakeholders as they negotiate, implement, and adapt new practices. Each construct comprises four corresponding components. Table 1 summarises the NPT constructs and illustrates how they were operationalised to guide the analysis.

**Table 1:** The four constructs of the Normalization Process Theory (NPT) are associated with the four corresponding components

Construct	Components	Explanation
*Coherence* Sense-making: does the intervention have a clear, shared rational?	Internalization	Perceived intrinsic value and purpose
	Individual specification	Personal meaning and scope of the pharmacist-clinician role
	Differentiation	Whether the role is distinguishable from existing practices
	Communal specification	Working together with others to build a shared understanding of the aim, objective, and expected benefits
*Cognitive participation* Effort: the relational work: Who will commit to, and drive, the intervention?	Initiation	Key participants in the initiation of pharmacist-clinicians’ practices
	Enrolment	Strategies used to engage others and to sustain engagement
	Legitimation	Ensuring that others believe it is right for them to be involved, and that they can make a valid contribution
	Activation	Defining actions and procedures needed to sustain pharmacist-clinicians’ practices
*Collective action* Commitment: the operational work: How does the intervention reconfigure everyday tasks and interactions?	Interactional workability	Impact on interactions, particularly the interactions between pharmacist-clinicians and other healthcare professionals
	Relational integration	Impact on relations between pharmacist-clinicians and (interprofessional) colleagues
	Skill set workability	Availability of the requisite competences
	Contextual integration	Provision of resources, time, and organisational support
*Reflexive monitoring* Appraisal: the appraisal work: Is the intervention delivering sufficient value to warrant its continuation?	Systematization	Formal monitoring and evaluation
	Communal appraisal	Collective assessment
	Individual appraisal	Personal assessment
	Reconfiguration	Adaptive modifications based on feedback

### Research team reflexivity

This study was conducted with an interdisciplinary team with backgrounds in community pharmacy, hospital pharmacy, medicine, and health sciences education research.

The pharmacists involved (KL, IW, TE) all reported self-experience in clinical roles, which may have influenced participant recruitment and the interpretation of the results. Because of their direct involvement with training of hospital pharmacists in hospital pharmacy in the Netherlands, TE and IW were excluded from the analysis of the raw data. They were only involved in the analysis of the pseudonymised data.

### Ethics approval

The study was approved on October 10th, 2024, by the Institutional Review Board (IRB) affiliated with the Department of Pharmacoepidemiology & Clinical Pharmacology, Department of Pharmaceutical Sciences, Faculty of Science, Utrecht University. (nr. UPF 2415).

All data were handled and stored according to the study’s data management protocol.

## Results

A total of 21 participants were interviewed, covering a broad range of work settings and levels of experience. Participants’ characteristics are summarized in Table 2.

**Table 2:** Table of study participant characteristics (n = 21)

Variable	n	%
Gender		
Male	6	28
Female	15	72
Type of hospital		
Academic	10	47
Teaching (non-academic)	9	43
Non-teaching	2	10
Working experience		
Ambulatory care pharmacist	2	10
Hospital pharmacist in training	2	10
Hospital pharmacist	17	80
Pharmacist-clinician tasks		
Consulting patients	11	53
Outpatient clinic in a hospital	7	33
Pharmacist on ward (working in clusters)	7	33
Bedside visits/Multidisciplinary consultation	11	53
Medication reviews	12	57

### Learning, adopting, and sustaining the role

Four main themes that cover the key factors that shape learning, adopting and sustaining the pharmacist-clinician role emerged from the experiences. These themes are presented in a chronological order reflecting how participants described the development of the pharmacist-clinician role in practice, from sense-making and adoption to enactment and sustainment.

#### Theme 1. Scope and role enactment of the pharmacist-clinician role

Participants differed substantially in how they understood and enacted the pharmacist-clinician role, particularly regarding its scope and the necessity of direct patient contact. These divergent views shaped early learning processes and participants’ efforts to establish the role’s legitimacy in practice.

Participants differed in which elements they included when describing the scope of pharmacist-clinician work. Across interviews, these elements included medication reviews, participation in ward rounds and multidisciplinary consultations, and consultations with patients and caregivers. Direct (face-to-face) patient contact was described as scarce and was not universally considered mandatory for the role. While some participants framed patient contact as a source of professional meaning and legitimacy, others emphasized its instrumental value rather than its centrality to the role.

‘You need to deliver good pharmaceutical care, and for that, it may be necessary to speak with patients. However, speaking with patients in itself is not the goal. It is a means.’ P06

Role enactment was primarily shaped at the local level rather than through institutional frameworks. Some participants described clinical activities as integral to their roles, whereas others experienced them as additional or exceptional. Participants also distinguished between different types of hospital pharmacists within pharmacy teams and emphasized the need for role differentiation to ensure effective organizational functioning:

‘There are pharmacists who are truly more back-office oriented, and that work also needs to be done. (…) You should not have those kinds of people running an outpatient clinic. So I think both types are needed for good organizational functioning.’ P15

Overall, these findings highlight the absence of a shared role definition for the pharmacist-clinician role and demonstrate substantial variation in expectations, activities, and sources of legitimacy across local contexts.

#### Theme 2. Interprofessional collaboration as the driver for adoption

Interprofessional collaboration was reported as the primary driver for adopting the pharmacist-clinician role, facilitating entry into clinical practice, legitimizing the role, and creating ongoing learning opportunities. Existing interprofessional collaboration often served as the initial starting point, inspired by existing networks or role models. Positive interprofessional relationships generated increased demand for pharmacist input, supported role expansion, and enhanced motivation, with network-building described as a core skill.

Several participants described collaboration with physicians as energizing and rewarding, contributing to increased visibility and recognition:

‘I receive a lot of questions from the <name> department, like: “I have this patient, what should I do?” So I have really become the go-to person for their department (…). So yes, I think visibility also plays a role there. I simply really enjoy working together with other professionals.’ P17

Overall, the adoption of the pharmacist-clinician role depended primarily on relational work and social positioning rather than formal role allocation.

#### Theme 3. Experiential workplace learning is more important than formal learning

Participants predominantly developed the pharmacist-clinician role through experiential, on-the-job learning, with competence developing through repeated enactment rather than formal training alone.

Participants highlighted interprofessional collaboration as an important learning opportunity for developing communication skills and gaining practical insight into clinical practice:

‘…Conversely, you also learn from others, you know? When I am in a multidisciplinary team meeting and they tell me about laboratory results and say: “well, with this patient you can clearly see from the electrolytes that they are completely dehydrated,” I think: ah yes, then next time I can think about that better as well.’ P12

Competence emerged through repeated exposure, trial-and-error, and feedback in real clinical settings, with hands-on experience considered pivotal for learning patient conversations. Formal education supported learning, but was insufficient on its own; participants valued communication skills, pro-activeness, organizational sensitivity, and interprofessional skills most.

‘You learn patient conversations by doing them a great deal. You do not learn them in a course. In a course, you can improve your skills, but you do not learn them.’ P04

Pharmaceutical knowledge was considered necessary, but rarely the limiting factor for enacting the pharmacist-clinician role. Participants differed in the importance they attributed to specialty knowledge; while some viewed it as essential in certain specialisms, others emphasized general knowledge over in-depth specialization:

‘The complex aspects of the ICU are things the intensivists themselves already know well. What they actually find important is to know all the other things from you. So in fact, my experience is that you know much more than you think yourself.’ P18

Overall, for learning and adopting the clinician role, learning the role in the workplace is pivotal.

#### Theme 4. Organisational conditions shape sustainment and scalability

While individual capability enabled the initial uptake of pharmacist-clinician work, participants consistently indicated that long-term sustainment depended on organisational conditions. This included protected time, adequate coverage and replacement, managerial and collegial support, and formal organisational recognition.

Several participants described proactively carving out time for pharmacist-clinician activities within their existing scope of duties, sometimes already during their residency period. The Entrustable Professional Activities (EPAs) -based residency program created opportunities to experiment with and legitimise pharmacist-clinician work during training. However, this did not guarantee sustainment post-qualification. Many initiatives remained fragile, person-dependent, and were diminished when initiators left or when protected time was withdrawn. As one participant illustrated:

‘When I had finished my residency, the outpatient clinic was simply gone—it just ceased to exist’ P06

A recurrent vulnerability concerned the lack of replacement or coverage, making pharmacist-clinician roles highly person-dependent. Participants noted that sustaining these roles often required task reorganisation within the pharmacy department, which was described as necessary but politically sensitive. Successfully implementing such changes depended on local power dynamics and managerial willingness to redistribute or reorganise work.

Another vulnerability related to the funding of pharmacist-clinician initiatives. As healthcare financing in the Netherlands remains strongly oriented towards reimbursing measurable procedures, clinical activities that do not translate into billable services were perceived as difficult to legitimise. Consequently, pharmacists experienced pressure to account for their work in procedural terms.

Although participants frequently experienced appreciation from colleagues and clinical teams, this recognition was largely informal and not structurally embedded. Several participants argued that this limited organisational commitment was partly driven by the difficulty of demonstrating and quantifying the value of pharmacist-clinician work in ways that aligned with managerial accountability frameworks:

‘When have you actually spent an hour usefully—say, when sitting in an multidiscliplinary team meeting… Do you then need to carry out a certain number of interventions to say, “this was a useful hour”? Or if you spend a whole hour without saying anything—well, is that useful or not?’ P19

Overall, while individual capability and motivation enabled the initiation of pharmacist-clinician activities, participants emphasized that organisational continuity ultimately determined their long-term sustainability.

#### A NPT-informed synthesis of the learning, adoption, and sustainment of the pharmacist-clinican role

**Fig. 1:**
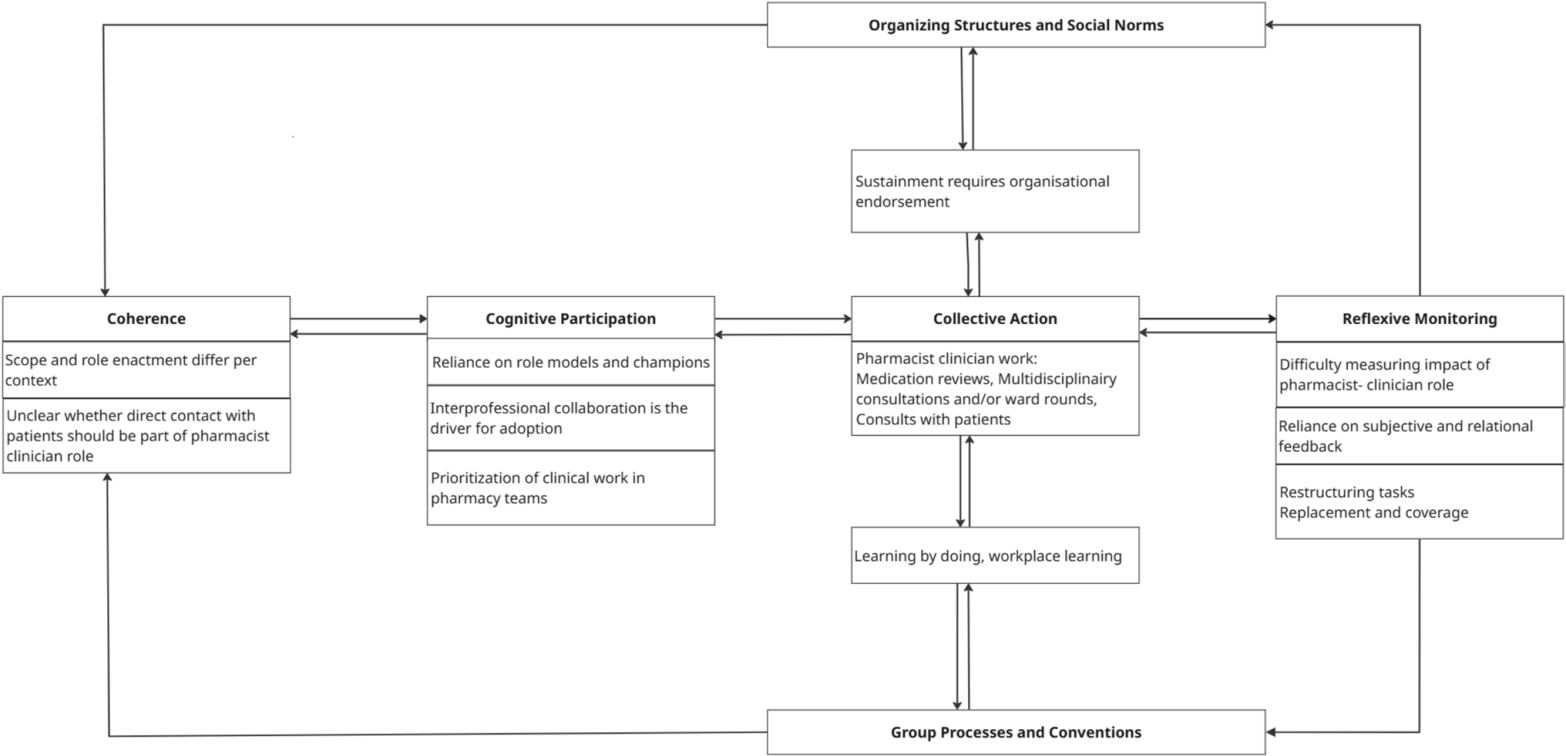
Overview of the study (sub)themes mapped to the four constructs of Normalization Process Theory (NPT), and how their interrelations influence the implementation and sustainment of the pharmacist-clinician role

While individual capability and motivation enabled the initiation of pharmacist–clinician activities, participants emphasized that organisational continuity and structural support ultimately determine whether these roles are sustained in routine practice. Sustainment of this role can, therefore, be understood as an innovation adoption process. We therefore drew on Normalization Process Theory (NPT) to further explore this process and synthesise how sense-making, engagement, enactment, and appraisal shaped both the adoption and sustainment of the role. Figure 1 illustrates how the study (sub)themes relate to the four NPT constructs, and Table 3 provides an overview of the NPT-informed synthesis of the findings.

**Table 3:** NPT-informed synthesis of findings

NPT construct	What participants described	What this explains
Coherence (sense-making)	Lack of shared understanding of role scope and the necessity of patient contact	Why learning and legitimacy varied across settings
Cognitive participation (engagement)	Reliance on role models, champions, and interprofessional allies, and prioritization of clinical work in pharmacy teams	How adoption was initiated and maintained locally
Collective action (enactment)	Learning-by-doing, task reorganization	Why competence developed through practice rather than training alone
Reflexive monitoring (appraisal)	Difficulty measuring value; reliance on subjective and relational feedback	Why sustainment required organisational endorsement

Adoption of pharmacist-clinician work across settings can largely be understood by using the NPT constructs of *cognitive participation*, *collective action*, and *reflexive monitoring.* Adoption was driven by intrinsically motivated individuals who possessed organisational sensitivity and networking skills, which enabled them to create opportunities for role enactment within existing structures. Adoption was further supported by role models, interprofessional allies, and local champions, as well as by managerial support, particularly through protected time and colleagues substituting and covering. Finally, positive feedback on the perceived value of pharmacist-clinician work reinforced continued engagement and legitimised early enactment of the role.

The NPT constructs of *coherence* and *reflexive monitoring* help explain why sustainment often remained fragile. Sustainment was hindered by limited collegial support within pharmacy teams and by the absence of shared understanding and objective ways to assess the value of pharmacist-clinician work. Moreover, a lack of shared understanding of the role’s scope and the necessity of direct patient contact contributed to interlocal variation in role enactment, thereby impeding organisational sense-making and constraining the role’s normalization.

## Discussion

### Summary of key findings

Early adopters had generally learned and adopted the pharmacist-clinician role through a combination of local interprofessional collaboration, experiential learning in daily practice, and individual initiative. Adoption was typically facilitated by existing professional relationships with other healthcare professionals and reinforced through learning-by-doing. These processes enabled pharmacists to enact the role and develop core competencies in the workplace. In contrast, long-term sustainability depended largely on organisational conditions. Participants consistently indicated that continuity beyond individual champions required formal arrangements such as protected time, coverage and replacement, managerial support, and explicit recognition. Although the benefits of the role were often visible to patients and interprofessional colleagues, this was not consistently translated into structural endorsement at the organisational level. Taken together, these findings highlight a critical distinction between aspects of the pharmacist-clinician role that can be supported through education and workplace learning, and those that require organisational and systemic action. This distinction provides a conceptual basis for understanding both the potential and the limits of educational interventions in strengthening the pharmacist-clinician role.

### Strengths and limitations

Several measures were taken to enhance the trustworthiness of this study. Reporting followed the COREQ checklist, supporting transparency in the design, conduct, and reporting of the study. The interview guide was pre-tested in four pilot interviews and refined before use, improving the clarity and relevance of the questions. Interviews were conducted by two researchers, which reduced dependence on a single interviewer and supported consistency in data collection. Trustworthiness was further strengthened through iterative analysis involving multiple researchers from different disciplinary backgrounds. Although thematic saturation appeared to have been reached after approximately 15–17 interviews, a small number of additional participants were included to complete scheduled interviews and ensure subgroup representation. In addition, participants were recruited from diverse hospital settings across the Netherlands, allowing us to capture variation in local implementation and organisational embedding. This detailed contextual description may support transferability to comparable hospital pharmacy settings beyond the Netherlands, although differences in healthcare systems and professional roles should be taken into account.

A limitation is that our findings mainly reflect the perspectives of pharmacists who supported the pharmacist-clinician role. Although some participants were no longer performing the role at the time of the interview, all remained supportive, which may have led to underrepresentation of resistance, disengagement, or de-implementation. We also did not interview organisational stakeholders, decision makers, patients, or interprofessional colleagues, limiting insight into broader perspectives on sustainment and perceived value.

### Interpretation

Clinical pharmacy practice is widely studied, yet much of the literature emphasises individual requirements rather than systemic and organisational requirements to embed and sustain the pharmacist-clinician role in routine care. Our findings add to earlier work that limited shared understanding of the role’s definition, scope, and responsibilities contributes to variation in enactment and complicates its embedding into everyday practice [4, 5, 22, 23]. This lack of shared understanding also extends to the meaning of direct patient care. This is illustrated by a recent Australian study showing that pharmacists spent over 80% of their work time performing clinical activities for patients, of which only around 12% was spent face-to-face with patients, suggesting that pharmacist-clinician work may often be delivered without direct patient contact [24].

Our findings indicate that while the pharmacist-clinician role is often initially taken up through individual initiative, workplace learning, and local interprofessional collaboration, its long-term sustainment depends largely on organisational conditions. Participants described how sustaining pharmacist -clinician activities required structural support, including protected time, clear role definitions, and organisational arrangements that integrate clinical work into routine practice.

Recent studies across different healthcare contexts support this interpretation. Conceptual work by Rushworth et al. has articulated the pharmacist-clinician role more explicitly as an autonomous and clinically integrated form of practice within collaborative care, positioning it as part of a broader transformation in healthcare systems that requires not only expanded competencies but also organisational and system-level change [25]. In a European survey of hospital pharmacy practice, limited capacity, low managerial priority, and perceptions that these activities are already undertaken by other healthcare professionals were among the barriers most frequently reported for the implementation of clinical pharmacy activities [12]. In South Africa, Crafford et al. examined barriers to service implementation through the lens of Self-Determination Theory and identified two overarching themes—time and trust—showing how the absence of formalised clinical pharmacist posts and limited understanding about the role constrained dedicated ward time and undermined both motivation and implementation. They also described how the lack of formal positions and a clear scope of practice created ongoing performance pressure to justify and document clinical work, reinforcing role ambiguity and resistance within and beyond pharmacy teams [26]. In Scotland, McLean et al. used Normalisation Process Theory to study advanced pharmacist prescribing and identified barriers to embedding the prescribing role across all four NPT constructs, including lack of shared understanding, insufficient infrastructure and appropriate roles, resource constraints, and limited strategic alignment and evaluation [23].

Together, these findings suggest that long-term sustainment depends not only on individual effort, but also on organisational prioritisation and structural embedding. Our findings also align closely with work on advanced practice nursing roles, which has highlighted the importance of clear role definitions and supportive organisational conditions for implementation and sustainment [27].

Our finding that learning occurs primarily on the job aligns with Eraut’s work [28] on informal workplace learning, which emphasises that much professional learning is embedded in everyday practice and often remains implicit. Participants’ accounts of developing patient consultation skills and enacting that role “by doing” illustrate how competence is built through repeated participative learning in daily work rather than through formal training alone. This underlines that strengthening the pharmacist-clinician role requires organisational and systemic conditions that enable workplace learning—particularly time, staffing and task allocation, and managerial support.

### Further research

This exploratory study focused on the adoption and implementation of the pharmacist-clinician role in the Netherlands. Future research in other countries and practice settings would help to examine how these findings translate across different healthcare systems and regulatory contexts. While the exact form of the role will depend on local structures and regulations, the mechanisms identified in this study are likely to be relevant across contexts.

Although recent work has begun to conceptualise the pharmacist-clinician role [25], a generally accepted definition and a concrete articulation of its scopes, tasks and responsibilities remain lacking across national and international contexts. Further research is therefore needed to refine the conceptualisation of the role and to inform subsequent policy development and evaluation. This should also include the perspectives of organisational and policy-level decision makers, as their views are likely to be important for understanding how the role is supported, formalised, and sustained in practice.

Our analysis highlighted the importance of organisational and systemic conditions for sustaining the role. NPT provided to be a useful framework for interpreting these processes. Future research could extend this work by incorporating NPT from the outset and further examining sustainment from this perspective.

Another essential element in training future pharmacist-clinicians is professional identity formation. Alongside facilitating and sustaining the role in practice, it is important that the professional identity of hospital pharmacists evolves in step with the development of the pharmacist-clinician role. We therefore recommend further research into how young hospital pharmacists and residents experience, interpret, and negotiate this role within their professional identity.

## Conclusion

Our findings indicate that a pivotal step in establishing a structural pharmacist-clinician role in institutes requires deliberate investment in implementation and sustainability, including the development of a clear role definition with concrete tasks and responsibilities. The pharmacist-clinician role should not be understood merely as an expansion of professional competencies, but as a partially implemented organisational innovation. The role exists at varying stages of development, shaped by local conditions and organisational arrangements, and requires more than the acquisition of clinical skills. Sustainable role development involves processes of learning, adoption, and embedding in everyday practice, supported by organisational allocation of time, legitimacy, and integration into workflows and responsibilities.

## Supplementary Information

Below is the link to the electronic supplementary material.

Supplementary file1 (DOCX 52 KB)

## Data Availability

The participants of this study did not give written consent for their data to be shared publicly, so due to the sensitive nature of the research, raw interview data are not available.
