# Supplementary material for: Learning, adopting, and sustaining the pharmacist-clinician role in clinical practice: a qualitative study among early adopters in the Netherlands
Source: Int J Clin Pharm. 2026 May 8;48(4):1672–81. doi: 10.1007/s11096-026-02150-y (PMC13369639; doi:10.1007/s11096-026-02150-y)
Supplement: Supplementary file 1 — Supplementary file1 (DOCX 52 KB) [file 11096_2026_2150_MOESM1_ESM.docx]

## Supplementary material Learning, adopting, and sustaining the pharmacist-clinician role in clinical practice: a qualitative study among early adopters in the Netherlands.

## APPENDIX 1 - Structured topic guide

**Main topics — Questions**

**Tasks in direct patient care**

- What types of tasks in direct patient care have you taken on?
  (Please give concrete examples, such as consultations or other activities, and explain.)
  How are these tasks organised, for example on the ward, in your own outpatient clinic, or otherwise?
- How often per month/week do you perform these tasks?
  Or how many hours do you spend on them? Do you work alone or, for example, in a joint role? Where do these activities take place (outside the pharmacy)?
- Is this sufficient to perform the task properly (and who or what determines that)?
  Do you see the patient directly? Where?
- What was the reason for taking on these tasks? How did this come about?
  Why did you start performing these tasks?

**Motivations**

- What motivates you to carry out these tasks?
- What does it bring you? For yourself, for others, for physicians, for professional goodwill?

**Competencies**

- Which competency/competencies have you developed or improved in order to perform your tasks as a treating clinician in direct patient contact?
  Which competencies are needed? What do you need to be able to do to perform this task well (how, when is it done properly)?
  (Knowledge, attitude, skills — how do you position yourself towards patients?)
- How did you develop or improve these competencies?
  (Courses, training, placements, practice, or otherwise?)
  (Probe for multiple competencies.)
- What do you think hospital pharmacy residents should learn during training in order to fulfil a similar clinician role?
  (Or what type of education do you envision — format, content, etc.?)
- Would you yourself have liked to learn this in training?
  Which competencies/knowledge/skills/attitudes would you have liked to acquire during residency?
  (And when, in what way, etc.)
  Or: what would you have liked to learn/practice/see more of during training?
- So if someone is good at a certain competency, does that mean they could also run this clinic?

**Organisational factors**

- Are there organisational factors that play a role in enabling you to perform the clinician role with direct patient contact? If yes, which ones?
  (For example support/recognition/reward from leadership, hospital mission/vision, or other factors.)
- Are there any disadvantages?
- What happens if you are not present (holiday, illness, or outside working hours)? What happens to the task/role then?
  (Does the work continue?)
- Suppose you moved to another hospital — what would happen to the outpatient clinic/consultation service/role you currently fulfil?

**Background information**

- Type of hospital:
- Gender identity:
- Subspecialisation:
- Number of years working as a hospital pharmacist:

**Closing question**

- If someone were to take over your position, how would you select the right person and how would you train or induct them?

## APPENDIX-2 Coding Tree

| 1.1 Understanding the pharmacist-clinician role |  |  |
| --- | --- | --- |
|  | View |  |
|  | Vision on pharmacist as clinician |  |
|  | Clinical tasks |  |
|  |  | Patient consultations |
|  |  | Participation in multidisciplinary meetings (MDO) |
|  |  | Interprofessional patient discussions / ward rounds |
|  |  | Medication review in multidisciplinary care |
|  |  | Own outpatient clinic patients |
|  |  | Explaining medication use to patients or representatives |
|  |  | Activities during day shifts |
|  |  | Other |
| 1.2 Building interprofessional collaboration as the engine for adoption |  |  |
|  | Initial triggers for starting the role |  |
|  | Interprofessional learning |  |
|  | Collaboration as motivator |  |
|  | Role models | Presence of role models |
|  |  | Absence of role models |
|  | Building interprofessional relationships |  |
|  | Interprofessional collaboration skills |  |
|  | Support from multidisciplinary partners |  |
| 1.3.Learning to enact the role through practice |  |  |
|  | building blocks competencies |  |
|  |  | Attitude |
|  |  | Being transparent about knowledge limits |
|  |  | Empathy |
|  |  | Enthusiasm |
|  |  | Flexibility |
|  |  | Dealing with uncertainty |
|  |  | Openness |
|  |  | Organisational sensitivity |
|  |  | Proactiveness |
|  |  | Courage and self-confidence |
|  | Knowledge |  |
|  |  | General pharmaceutical knowledge |
|  |  | Immediate knowledge |
|  |  | No need for immediate knowledge |
|  | Experience |  |
|  |  | Practical experience |
|  |  | Specialist knowledge |
|  | Skills |  |
|  | Communication |  |
|  |  | With patients |
|  |  | Communication with professionals |
|  |  | Clinical reasoning |
|  |  | Organisation and time management |
|  |  | Problem Solving |
|  | Collaboration |  |
|  |  | Intraprofessional |
|  |  | Self-reflection and learning ability |
|  | How competencies are learned  Learning approaches |  |
|  |  | Tailoring learning to personality |
|  |  | Conferences |
|  |  | Courses |
|  |  | Learning through practice (by doing) |
|  |  | Self directed learning |
|  |  | Being open for feedback |
|  |  | Learning guidelines |
|  | Supervision |  |
|  |  | Role supervisor |
|  |  | Role of the training program |
|  |  | Unboarding new residents [AIOS] |
|  |  | Gradual reduction of supervision |
|  | Motivation |  |
|  |  | Contact with patients |
|  |  | Developing expertise |
|  |  | Understanding the full patient picture |
|  |  | Interest in the field |
|  |  | Intrinsic motivation |
|  |  | Learning new things |
|  |  | Enjoyment |
|  |  | Curiosity |
|  |  | Teaching |
|  |  | Research |
|  |  | Problem solving |
|  |  | Intellectual challenge |
|  |  | Responsibility |
|  |  | Sense of fulfilment |
|  |  | Recognition and appreciation |
| 1.4 Organisational conditions shape sustainment and scalability |  |  |
|  | Barriers |  |
|  |  | Small hospital |
|  |  | Need for sufficient patient volume |
|  | Colleagues |  |
|  |  | Organizing work processes more efficiently |
|  |  | Disadvantages of current working methods |
|  |  | Evidence for usefulness |
|  |  | Demonstrating added value |
|  | External outcomes for patients, the hospital, and the professional group. |  |
|  |  | Benefit for patients |
|  |  | Added value alongside physicians |
|  |  | Improved efficiency |
|  |  | Improved quality of care |
|  |  | Visibility and recognition of the specialty |
|  | Financial aspects |  |
|  |  | Reimbursement / declarations |
|  | ICT tools |  |
|  | “Something is better than nothing” approach |  |
|  | Maintaining role for training purposes (EPA1) |  |
|  | Embedding in organisation |  |
|  |  |  |
|  |  |  |
|  | initiator |  |
|  |  | Initiators / champions |
|  |  | Pilot initiatives |
|  | Governance and external actors |  |
|  |  | Hospital management |
|  |  | NVZA |
|  |  | Other organisational factors |
|  | Structural conditions | Outpatient care |
|  |  | Care pathways |
|  |  | Time allocation for tasks |
|  |  | Time availability and prioritisation |
|  |  |  |
| Participant characteristics |  |  |
|  | Area of expertise |  |
|  | Years of experience as hospital pharmacist |  |
|  |  | 0-9 years |
|  |  | 10-19 years |
|  |  | >20 years |
|  |  | Resident |
|  | Outpatient pharmacist |  |
|  | Gender |  |
|  |  | Male |
|  |  | Female |
|  | Hospital type |  |
|  |  | Academic hospital |
|  |  | Peripheral hospital |
|  |  | Top clinical hospital |
| Illustrative quotes |  |  |

## APPENDIX 3- Detailed overview of study findings, including NPT constructs, themes, and illustrative quotes

|  |  |  |  |  |
| --- | --- | --- | --- | --- |
| **NPT construct** | **NPT explanation** | **Example quotes** | **Themes and subthemes** | **Description** |
| **Coherence** | **Sense-making** |  |  |  |
| *Individual specification* | *Personal meaning and scope* | ‘You need to deliver good pharmaceutical care, and for that it may be necessary to speak with patients. However, speaking with patients in itself is not the goal. It is a means.’ P06 | *Scope of the pharmacist clinician role* | Unclear whether pharmacist-clinician role is part of standard HP job, and whether direct contact with patients should be part of the role |
| *Communal specification* | *Working together with others to build a shared understanding of the aim, objective, and expected benefits* | ‘If you look at the number of hospital pharmacists who actually have structural patient contact, I think there are, in the Netherlands, (…) not very many at all. So I think that if you have structural patient contact, you are already in a somewhat special position. There are, for example, some hospital pharmacists who work in the outpatient pharmacy. (…) They do, of course, have patient contact, but that is because they work in the outpatient pharmacy. Hospital pharmacists with a clinic-based role… well, fortunately there are more and more of those, so nationwide there are really many initiatives. (…) But so far, compared with the rest of hospital pharmacists, they simply remain scarce.’ P1 | *Role enactment* | Unclear whether pharmacist-clinician role is part of standard HP job, and whether direct contact with patients should be part of the role |
| *Differentiation* | *Whether the role is distinguishable from existing practices* | ‘Well, for me, those are activities that are part of my task package, so for me, I do not experience them as additional activities.’ P7  ‘We have also discussed that issue extensively. I would like it if everyone wanted that, but that is not realistic. There are also genuine back-office tasks. There are pharmacists who are truly more back-office oriented, and that work also needs to be done. For example, at <hospital name> we have people who really flourish when they can compound medicines and produce biotech, high-tech medicinal products—people who make that their life’s work. You should not have those kinds of people running an outpatient clinic. So I think both types are needed for good organisational functioning.’ P15 | *Role enactment* | Unclear whether pharmacist-clinician role is part of standard HP job, and whether direct contact with patients should be part of the role |
| *Internalization* | Perceived intrinsic value and purpose | ‘I find clinical patient contact valuable, because it often gives you a better understanding of what is going on with a patient.’ P03  ‘I think what I have always found very important is challenge. I do not like routine, and I think that here you could really go back into the content and delve deeper again.’ P17 | *Appreciation* | Direct patient contact is rewarding |
|  |  |  |  |  |
| **Cognitive participation** | **The effort of the people involved** |  |  |  |
| *initiation* | *Key participants in the initiation of pharmacist-clinicians' practices* | ‘Well, what intrigued me was, of course, <name of colleague hospital pharmacist>. They ran a clinic for patients with kidney failure. And, well, it was very much about being a treating professional and about being more on the ward or more present, and working together with the physicians.’ P14  ‘Because my colleague had done that, they had already paved the way, so to speak. (…) When I started, the path was already paved, as it were.’ P06  ‘That created a fertile ground, so those examples. I also had role models for how direct patient care can be delivered, and I am also really—well— inherently… P15 | *Organizational conditions and on the job learning* | Role models serve as important sources of inspiration and example, though they are scarce. |
| *Activation* | *Defining actions and procedures needed to sustain pharmacist-clinicians’ practices* | ‘That is, I think, primarily within the multidisciplinary field: clearly taking up your role within that, and demonstrating that you are an equal conversation partner—someone who knows the content they are talking about, but who also has an eye for the other disciplines and sufficient knowledge about the conditions the patient has. That is one aspect (…) and the other is (…) that over time you really do have to prove that you add value there, and that you genuinely have to find it interesting yourself to add value in that way.’ P07 | *Interprofessional collaboration - positioning* | Interprofessional collaboration facilitates and strengthens pharmacist-clinician initiatives.  These collaborations are also a source of learning opportunities, inspiration, and motivation. |
| *Enrollment* | Strategies used to engage others and to sustain engagement | ‘I have also discussed this with colleagues, with nurse practitioners I know—about how you do that, how you approach such a conversation with a patient?’ P11  ‘…Conversely, you also learn from others, you know? When I am in a multidisciplinary team meeting and they tell me about laboratory results and say, “well, with this patient you can clearly see from the electrolytes that they are completely dehydrated,” I think: ah yes, then next time I can think about that better as well.’ P12 | *Interprofessional collaboration – source for learning* | Interprofessional collaboration facilitates and strengthens pharmacist-clinician initiatives.  These collaborations are also a source of learning opportunities, inspiration, and motivation. |
|  |  | ‘That you therefore have commitment from your team of colleagues—like, “this is something we find important; this is where we see added value.‘ P03  ‘It is simply very good that we do this as a team, which means it is always covered. So if the ICU calls the acute care phone, it is always answered.’ P18  ‘Absolutely. Both from myself, you know, because I have to be able to plan it into my days and make room for it. But also certainly from your professional group. Because if others do not see the added value of you going to the ICU on a weekly basis, then it will not get off the ground. (…) In that case, it may be better (…) to demonstrate your added value in other ways. Because if you do not get that support from the team, it takes a great deal of time’. P12 | *Organisational conditions* | Intra-professional colleagues facilitate workload division and the sustainability of pharmacist-clinician work.  In some hospital pharmacies, there is no replacement for the pharmacist-clinician; in others, the work is divided among multiple colleagues. |
| *Legitimation* | *Ensuring that other participants believe it is right for them to be involved, and can make a valid contribution* | ‘No one can take over from me. No, no, no. So we need to do something about that.’ P13  ‘There is no replacement, so in principle, if I were to get hit by a bus tomorrow, it would simply be over. There is no replacement, no.’P01  ‘…a specialist outpatient clinic. We had that embedded for a while, but it was also part of a colleague’s PhD project. That colleague left, and then it was: they did not want to pay for it, so we stopped.’ P11  ‘So it can be done, but you also have to handle it politically in the right way. (…) If you believe in it, you will get there, but that was not the case at <hospital>, unfortunately.’ P14 | *Organisational conditions- sustainability* | Participants say that pharmacist-clinician work is not always supported by hospital (pharmacy) management and therefore not sustainably embedded. |
|  |  |  |  |  |
| **Collective action** | **Commitment** |  |  |  |
| *Skill set workability* | *Availability of the requisite competences* | ‘I think you do need a certain amount of knowledge. So yes, I think you have to make sure you have some readily available knowledge.’ P13  ‘Of course, you can retrieve a lot of knowledge from a particular book or source, but you need to be able to fit that into how it makes sense when thought through from the clinical reasoning of a treating professional.’ P10  ‘You learn patient conversations by doing them a great deal. You do not learn them in a course. In a course, you can improve your skills, but you do not learn them in a course, in my view.’ P04  ‘What I do think is that it is more a matter of experience, something like saying: “well, I have seen this so many times now; this is always the case,” or “I know that this is a really unpleasant drink,” or…’P19  ‘You acquire the knowledge during your training, but ultimately you have to apply it all in practice, and that is where you learn the most.’ P 21  ‘...The complex aspects of the ICU are things the intensivists themselves already know well. What they actually find important is to know all the other things from you. So in fact, my experience is that you know much more than you think yourself.’ P18  ‘It is not that you do not need knowledge, but I do not mean that you first have to attend psychiatric conferences very often, or that you have to keep delving into the literature endlessly before you go onto the ward (…). So for them, your added value really lies in that co-medication, which they know less about.’ P11  ‘Being able to listen well (…) to both patients and other healthcare professionals’ P08  ‘That knowledge can be learned. It is much more about the other competencies (…) that enable you to make a connection with the treatment team and with the patient.’ P15  ‘Yes, flexible, but also culturally sensitive, you know—because of course every hospital and every organisation has its own culture. So that you know how things work there, as they say.’ P08 | *Learning the pharmacist-clinician role* | Apart from a few very specialized functions, basic pharmaceutical knowledge suffices for most pharmacist-clinician work.  Most important are communicative skills and interprofessional skills (for networking and assessing patients’ needs)  Pro-active attitude that reflects the organisation’s needs. |
| *Skill set workability* | Availability of the requisite competences | ‘I have had a relationship with the people who work there for a much longer time, so for me it is also easy to be there’ P05  ‘It is also, in a way, about visibility and building relationships—what you are doing.’ P15  ‘It is absolutely about building a relationship. Yes, you do always have to prove yourself a bit, I think .’ P17 | *Interprofessional collaboration - networks* | Existing relationships facilitate new initiatives |
| *Contextual integration* | *Provision of resources, time, and organisational support* | ‘In a peripheral centre, you simply work with far fewer people, but you still have to do largely the same work. You have to have everything organised, even if it is for fewer patients. As a result, you are much more of a generalist in a peripheral centre and generally have little time to specialise in a specific population and to spend a great deal of time on that.’ P 20  ‘Would you like to do this more? Yes, I also think it is useful, but—this may sound a bit trivial—there is simply no time or capacity for it at the moment. Also when it comes to organising those MDT meetings, in addition to the regular MDTs that, for example, geriatrics already has. Because they are a kind of additional patients—you are essentially doing something extra—and that is extremely challenging to organise.’ P03  ‘Well, that mainly has to do with how we have organised the work in the hospital pharmacy. So you have to create that space, because at the moment the daytime schedule is full when you are on day duty. Then you cannot simply step away to speak with patients, because the time—the hours—are not there. So you would have to organise this differently, by doing it on days when you do not have day duty and are doing other tasks.’ P04 | *Organisational conditions - Support* | Time and space for pharmacist-clinician activities differ per training centre. |
| *contextual integration* | *Provision of resources, time, and organisational support* | ‘So there are a number of residents—those in training to become hospital pharmacists with us—who mainly do this to further develop EPA 1, because consultation skills and contact with patients are of course also part of that’ P09  ‘Yes, that also depends somewhat on how much space I could get for this within the training programme, and on what the department’s preference was.’ P12 | Organisational conditions | During their residency, HP experience time to set up pharmacist-clinician initiatives.  Additionally, the existence of EPA 1, 2, and 4 contributes to the deployment of pharmacist-clinician tasks. |
| *Interactional workability* | *Impact on interactions* | ‘It involved a redistribution of tasks, and within that redistribution of tasks it is also important to stop doing things that are no longer necessary for hospital pharmacists.’ P18  ‘And I do notice that this is a barrier for many other hospitals, but you can also reframe it and think, “hey, we are actually quite free to shape this care in the way we want.” We impose on ourselves that we have to perform medication surveillance every day. Perhaps we could organise this differently for our high-risk patients and use that time to deliver care in another way.’ P07  ‘So we do not have a classic day shift for patient care, but we work with clusters. In this system, each hospital pharmacist and/or clinical pharmacologist has a cluster consisting of a number of specialties, and you essentially work within that cluster every day. In the morning, you are also present on the ward, where the patients, physicians, and nursing staff are—for example, to join ward rounds, MDT meetings, or similar activities.’ P02  ‘Yes, I sometimes join the ward round, and the ward round takes place at the bedside, so we go along the patients. So if necessary, I do ask patients a question myself.’ P11  ‘But what I do in direct patient care is that, at the very least, I have two fixed outpatient clinics every week, where I see patients with kidney failure’ P07 | *Organisational conditions- Restructuring tasks* | Restructuring pharmacist tasks, prioritizing pharmacist-clinician work, and/or discontinuing obsolete tasks |
| *interactional workability* | Impact on interactions | ‘In fact, you mainly learn this in practice, and certainly through the training here in the hospital or in another hospital. That is where you really learn those competencies—what does and does not work.’ P03  ‘You learn patient conversations by doing them a great deal. You do not learn them in a course. In a course, you can improve your skills, but you do not learn them in a course, in my view.’ P04  ‘But you do need that experience to be able to perform the role properly. So in my view, the amount of hands-on experience you need is often underestimated to reach a certain level of competence.’ P10  ‘You acquire the knowledge during your training, but ultimately you have to apply it all in practice, and that is where you learn the most.’ P 21 | *Individual needs-*  *Exposure* | Learning on the job. |
|  |  |  |  |  |
| **Reflexive Monitoring** |  |  |  |  |
| *systematization* | *Formal monitoring and evaluation* | ‘Why do we as pharmacists think we have to justify and measure all of this? And in fact, there is no one who says, “Well, what are you actually doing here?”’ P019  ‘Then we conducted a pilot, in which, I believe, for three months I systematically recorded all the interventions I carried out, all consultation calls, and all other activities’ P11  ‘That is also to demonstrate what the added value actually is, because of course it does take quite some time—expensive time, you know.’ P17  ‘You can never fully prove it, but you can show where things are not going well and that this is a potential solution.’ P18  ‘We have also had extensive discussions about that. About, well, for example: when have you actually spent an hour usefully—say, when sitting in an MDT meeting? Do you then need to carry out a certain number of interventions to say, “this was a useful hour”? Or if you spend a whole hour without saying anything—well, is that useful or not?’ P19 | Organisational conditions -effectiveness | Unclear whether and how the value of pharmacist-clinician work should be measured. Hard end points (hospital admissions/ death) vs softer endpoints like wellbeing, fewer side effects, etc. |
| *Individual appraisal* | *Personal assessment* | ‘For me, the satisfaction really lies in concretely contributing to the improvement of that therapy for those people—so that you genuinely think: yes, this is something they benefit from.’ P08  ‘That feels good to me. Well, then you get very direct… you have a grateful patient. Well, that is very rewarding. If you have spent ten minutes and afterwards someone says, “this was very nice, thank you.” Just like a physician can really appreciate it when you have thought along for a moment, or identified an interaction, or looked something up—so yes, that is very rewarding to do’ P06  ‘It gives me job satisfaction.’ P19  ‘It also gives me a great sense of fulfilment.’ P01 | *Individual needs -Value* | As pharmacist-clinicians see the value of their contributions to patient care, it leads to fulfilment. |
| *communal appraisal* | *Collective assessment* | ‘Because we think—and have also seen in recent years—that it has an effect on various strategic priorities of the hospital, or that we can contribute to those in this way. And I think it is very important that the hospital clearly knows who you are, what you do, and why you do it. In doing so, you also strengthen your position.’ P2  ‘In the beginning, there were also a number of people who were somewhat hesitant, questioning what the added value actually was. But in the end, they have become the strongest advocates. That is something I have learned.’ P07 | *Organisational support- Appraisal* | pharmacist-clinicians feel being appraised by patients and IP colleagues. |
| *Reconfiguration* | *Adaptive modifications based on feedback* | ‘We chose to save a great deal of time on medication surveillance by, for example, implementing robust clinical rules, which means that we no longer have to perform about 90% of our interaction monitoring. We use that time to deliver direct patient care, but that is our own way of working. So we then say: in line with the hospital’s strategic priorities, we allocate a certain number of FTEs to patient care in order to deliver that care properly.  And I do notice that this is a barrier for many other hospitals, but you can also reframe it and think, “hey, we are actually quite free to shape this care in the way we want.” We impose on ourselves that we have to carry out medication surveillance every day. Perhaps we could organise this differently for our high-risk patients and use that time to deliver care in another way.’ P07  ‘It involved a redistribution of tasks, and within that redistribution it is also important to stop doing activities that are no longer necessary for hospital pharmacists.’ P18 | *Organisational conditions- intra-professional collaboration* | Reorganization of responsibilities |
